# Selection of a Clinical Lead TCR Targeting Alpha-Fetoprotein-Positive Liver Cancer Based on a Balance of Risk and Benefit

**DOI:** 10.3389/fimmu.2020.00623

**Published:** 2020-04-27

**Authors:** Xiaobing Luo, Huijuan Cui, Lun Cai, Wei Zhu, Wei-Chih Yang, Michael Patrick, Shigui Zhu, Jiaqi Huang, Xin Yao, Yihong Yao, Yukai He, Yun Ji

**Affiliations:** ^1^Cellular Biomedicine Group Inc., Gaithersburg, MD, United States; ^2^Georgia Cancer Center, Medical College of Georgia, Augusta, GA, United States; ^3^CodexSage LLC., Germantown, MD, United States; ^4^Department of Medicine, Medical College of Georgia, Augusta University, Augusta, GA, United States

**Keywords:** T cell receptor (TCR), hepatocellular carcinoma (HCC), alpha-fetoprotein (AFP), cross-reactivity, immunotherapy, alloreactivity, X-scan

## Abstract

Hepatocellular carcinoma (HCC) is the most common type of primary liver cancer with a poor prognosis and limited therapeutic options. Alpha-fetoprotein (AFP), an established clinical biomarker of HCC, has been employed as an attractive target for T cell-based immunotherapy against this disease given its high expression in the tumor and restricted expression in normal tissues. We have identified a number of T cell receptors (TCRs) recognizing the HLA-A^*^02:01 restricted AFP_158−166_ peptide FMNKFIYEI, providing a TCR candidate pool for identifying TCRs with optimal clinical benefit. To select the ideal AFP TCR for clinical use, we evaluated the efficacy and safety profile of 7 TCRs by testing their potency toward AFP-expressing HCC cells and their specificity based upon reactivity to normal and transformed cells covering a wide variety of primary cell types and HLA serotypes. Furthermore, we assessed their cross-reactivity to potential protein candidates in the human genome by an extensive alanine scan (X-scan). We first selected three TCR candidates based on the *in vitro* anti-tumor activity. Next we eliminated two potential cross-reactive TCRs based on their reactivity against normal and transformed cells covering a variety of primary cell types and HLA serotypes, respectively. We then excluded the potential cross-reactivity of the selected TCR with a protein candidate identified by X-scan. At present we have selected an AFP TCR with the optimal affinity, function, and safety profile, bearing properties that are expected to allow AFP TCR redirected T cells to specifically differentiate between AFP levels on tumor and normal tissues. An early phase clinical trial using T cells transduced with this TCR to treat HCC patients (NCT03971747) has been initiated.

## Introduction

Hepatocellular carcinoma (HCC) is the most common type of primary liver cancer, accounting for 75–85% of all liver cancers (1). There are about 841,000 new cases and 782,000 deaths annually caused by liver cancer (1). Liver cancer ranks fifth in terms of global cancer cases and second in terms of cancer death in males (1). In China, there are ~466,000 cases of liver cancer diagnosed annually, with the mortality reaching 422,000 each year (2), more than half of the world rate. HCC in adults is often diagnosed in later stages. Current treatments of HCC are limited to surgery, local ablation, liver transplantation, and targeted therapies, which show restricted efficacy and minimal survival benefits for the majority patients (3). New therapeutic means are urgently needed to effectively treat this malignancy. Among them, T cell-based immunotherapy appears to be a promising clinical intervention for HCC patients due to the following observations. First, it is found that there is a correlation of improved overall survival rate with the number of CD8^+^ T cells infiltrated into HCC (4, 5). Second, the down-regulation of HLA-class I expression in most solid tumors generally does not occur in HCC (6). Instead, HLA-A is upregulated in more than 50% of the patients (6).

T cell-based immunotherapies have proven to be one of the most potent ways to treat late-stage malignancies, especially B-lineage hematologic malignancies (7–9). Efficacy of T cell-based immunotherapies against solid tumors, however, are still limited due to lack of optimal tumor specific targets, heterogeneity of tumor antigen expression, poor persistence of transferred cells, and immunosuppressive tumor microenvironment (7–10). Among these factors, lack of tumor-specific targets is a major obstacle, limiting the effectiveness and safety of T cell-based immunotherapies (9, 11, 12). Therefore, new immunotherapies targeting a HCC specific antigen holds promise to improve the treatment of this devastating disease.

Alpha-fetoprotein (AFP), a secreted 70 kD glycoprotein, has been used as a biomarker for HCC as elevated expression of AFP in tumors and serum is found in 60–80% of HCC patients and correlates with poor prognosis (13). AFP is commonly expressed in the fetal liver and yolk sac during the first trimester of pregnancy, but declines sharply after birth and remains low in adulthood (13). While vital for the developing fetus, the role of AFP in adult tissues is less well-understood but appears dispensable, thus making it a promising tumor antigen for T cell-based immunotherapies (13). A human TCR specific for AFP_158−166_ was recently identified from healthy donors, but the antitumor effect is restricted, probably due to its low affinity (14). Recently, TCR (15) (NCT03132792) and TCR-mimic CAR (16) (NCT03349255) developed to target AFP expressing HCC have been employed in clinical trials. To date, the safety profile remains good, and promising signs of clinical efficacy have been observed (17, 18), suggesting that AFP is a good target for T cell-based immunotherapies.

We have identified a number of TCRs against AFP_158−166_ from HLA-A2 transgenic AAD mice with varied affinity using a lentivector-prime and peptide-boost approach (19). To select the most optimal AFP TCR for clinical use, potent anti-tumor activity would need to be achieved while avoiding severe off-tumor toxicity previously observed in a few clinical trials (12, 20–23). To accomplish this, we first performed serials of *in vitro* assays to select TCRs with potent activity against AFP-expressing tumor cells. Next we evaluated the safety profile of the three selected TCRs by testing the TCR expressing cells against normal and transformed cells, which include a variety of primary cell types and HLA serotypes, respectively. In addition, our colleagues [accompanied study, (24)] performed an X-scan screening to exclude the potential cross-reactivity of TCR 1-3 with other protein candidates in the human genome. We further confirmed that the selected TCR did not cross-react with the potential candidate with serials of validation assays. Based on these analyses, we have selected a TCR based on the balance of its activity and safety profile. This AFP TCR bears properties that are expected to allow T cells, redirected with this TCR, to specifically differentiate between AFP levels on tumor and normal tissues. An early phase clinical trial using T cells transduced with this TCR to treat HCC patients (NCT03971747) has been initiated.

## Materials and Methods

### TCR Cloning

For each TCR, the coding sequences of its α and β chain were codon-optimized, joined with a P2A linker, and cloned into a lentiviral backbone under the EF1α promoter.

### Lentivirus Production

For packaging, 293T cells (ATCC) were seeded in poly-L-Lysine coated plates (Corning) and transfected the next day with the mix of AFP TCR transfer plasmid and 3 packaging/envelope plasmids, using lipofectamine 3000 (Thermo Fisher). Forty-eight hours after transfection, the virus-containing media were harvested and centrifuged to remove cell debris. The virus supernatant was then directly used for transduction or immediately stored at −80°C.

### Generation of AFP TCR-T Cells

Peripheral blood mononuclear cells from healthy donors were obtained from Precision for Medicine (Fredrick, MD). Total or CD8^+^ T cells were isolated using either EasySep™ Human T Cell Isolation Kit or EasySep™ Human CD8^+^ T Cell Isolation Kit (both from StemCell Technologies), respectively, following the manufacturer's protocol. The isolated cells were then cultured in AIM V medium (Thermo Fisher) supplemented with 10% fetal bovine serum (FBS; VWR) and 200 IU/mL IL-2 (Peprotech), along with Dynabeads™ Human T-Activator CD3/CD28 (Thermo Fisher; cell to bead ratio 1:1). After 24 h of activation, cells were transduced with AFP TCR lentivirus in the presence of 10 μg/mL Protamine Sulfate (Sigma). The transduced cells were expanded for 9–11 days and then used for downstream analysis or cryopreserved with Cryostor D10 media (Biolife Solutions).

### Cell Lines, Primary Cells, and iCells

HepG2 and Huh7 cells were obtained from ATCC. MDA-MB231 cells were obtained from Dr. Hasan Korkaya who originally purchased from ATCC. All cell lines were maintained in DMEM medium supplemented with 10% FBS (VWR). The Epstein-Barr virus (EBV)–transformed B-lymphoblastoid cell lines (B-LCL) used for alloreactivity test were obtained from either Sigma or Fred Hutchinson Cancer Research Center, and maintained in RPMI 1640 medium supplemented with 15% FBS (VWR). Primary adult human hepatocytes were obtained from Lonza. Primary human lung and kidney epithelial cells were purchased from Novabiosis and Lifeline, respectively. Induced pluripotent stem cell-derived iCell® Neurons, Astrocytes, Cardiomyocytes and Endothelial Cells were all from FUJIFILM Cellular Dynamics, Inc. The culture medium, supplements and plate coating reagents for each cell type were purchased from vendors as designated by the cell suppliers. Primary hepatocytes and the four iCells were originally from HLA-A^*^02:01^+^ donors, according to vendor-provided information. Primary lung and kidney epithelial cells were originally from HLA-A2^+^ donors, in-house PCR (25) and sequencing further confirmed they also carry HLA-A^*^02:01 allele.

### Co-culture

HepG2, Huh7, and B-LCL cells were harvested and resuspended in IL-2 free T cell medium. Total 50,000 cells were then seeded in each well of a 96-well plate, followed by addition of equal number of AFP TCR T or untransduced control T cells. After overnight co-culture, the supernatant was saved for ELISA, and cells from replicate wells were combined for FACS analysis of 4-1BB activation wherever indicated. Primary lung and kidney epithelial cells and iCell Endothelial Cells were expanded after thawing, and the 2nd passage cells were harvested for co-culture with T cells as stated above. Co-culture with other primary cells or iCells was done similarly except that primary hepatocytes, iCell Neurons, Astrocytes or Cardiomyocytes were seeded in 96-well plates immediately after thawing, and T cells were added the next day for overnight incubation.

### IFN-γ ELISA

The ELISA plates were coated with a human IFN**-**γ monoclonal capture antibody (Thermo Fisher, cat. M700A) overnight at 4°C. After washing and blocking with assay buffer, cell culture supernatants or diluted IFN**-**γ standards (Biolegend, cat. 570209) were added together with the biotin-labeled IFN**-**γ antibody (Thermo Fisher, cat. M701B) and incubated at room temperature for 1.5 h. HRP-conjugated Streptavidin (Thermo Fisher, cat. N100) and TMB Substrate (Thermo Fisher, cat. 34021) was then sequentially added for detection. After stopping the reaction, the absorbance was measured at 450 nm by a SpectraMaX iD3 plate reader (Molecular Device).

### FACS

Cells were first stained with LIVE/DEAD™ Fixable Aqua (Thermo Fisher, cat. L34957), followed by staining with antibodies against various surface markers. Samples were then acquired by a FACS instrument (CytoFLEX LX, Beckman Coulter). For intracellular staining, GolgiPlug (BD) was added upon at the beginning of T cell and target cell co-culture and cells were cultured for 6 h. Samples were then processed with the Cytofix/cytoperm kit (BD, cat. 555028) following the manufacture's protocol. The following antibodies were used: APC-eFluor 780 anti-human CD3 (Thermo Fisher, cat. 47003642), FITC anti-human CD8a (Biolegend, cat. 301006), BV605 anti-mouse TCRβ (Biolegend, cat. 109241), APC anti-human CD137 (Biolegend, cat. 309810), PECy7 anti-human IFN-γ (Biolegend, cat. 502528), Alx700 anti-human TNF-α (Biolegend, cat. 502928), PE anti-human IL2 (Biolegend, cat. 500307), PerCPCy5.5 anti-human CD107a (Biolegend, cat. 328616). HLA-A2/AFP158 tetramer was kindly provided by NIH Tetramer core facility at Emory University. All data were analyzed with FlowJo V10 software (FlowJo).

### Cytolytic Assay

HepG2 cells were seeded in the 96-well E-plate (30,000 cells/well) and placed in the Real-time Cell Analyzer (RTCA MP, ACEA Biosciences) to record the cell index. The next day T cells were added at the indicated effector to target ratio, and cell index was recorded continuously for another 4 days. The cell index curve was then normalized to the time point just before adding T cells, and transformed as percentage cytolysis by comparing the average of triplicate in each T cell group with that of target cell only group (i.e., only T cell medium was added).

### Western Blot

Cell lysates were extracted with RIPA buffer (Thermo Fisher), separated on a 4–12% SDS-page gel (Thermo Fisher), and transferred onto nitrocellulose membrane using iBlot 2 gel transfer device (Thermo Fisher). The membrane was blotted with indicated primary antibodies. After incubating with corresponding secondary antibody, signals were detected on an Odyssey Fc imaging system (Licor). ENPP1 antibody was purchased from Abcam (cat. ab223268). AFP and GAPDH antibodies were from Santa Cruz Biotechnology (cat. sc-8399 and sc-47724, respectively). IRDye® 800CW Goat anti-Rabbit IgG and IRDye® 680RD Goat anti-Mouse IgG secondary antibodies were from Licor.

### Bead-Based Immunoassay

The LEGENDplex™ Human CD8/NK panel (BioLegend, cat. 740267) was used for simultaneous quantification of 13 soluble analytes in cell culture supernatants, following the manufacture's protocol. The assay was read on a CytoFLEX LX FACS instrument (Beckman Coulter).

### Statistical Analysis

Data were analyzed using the GraphPad Prism software (Version 5). Unpaired Student *t*-test was conducted for comparison and two-tailed *P* < 0.05 was considered statistical significant.

## Results

### Screening AFP TCRs for Their Anti-tumor Activities *in vitro*

**Figure 1:**
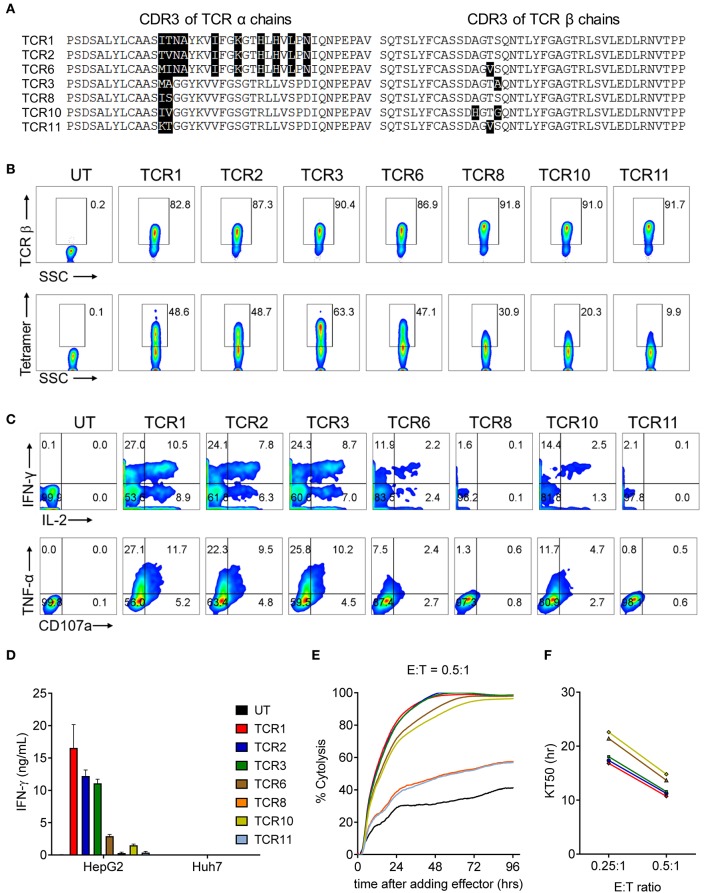
AFP TCR1, 2, and 3 demonstrate comparable potent reactivity toward the target cells *in vitro*. **(A)** Alignment of amino acid sequence of CDR3 regions of the α (left) and β (right) chains of the 7 AFP TCRs. **(B)** FACS analysis of the surface expression of TCR β chain and AFP tetramer staining of T cells 8 days after transducing of indicated AFP TCRs. The percentage of TCR β (top) or AFP tetramer (bottom) positive cells as indicated. **(C)** Cytokine production and degranulation of AFP TCR T cells upon encountering HepG2 target cells as revealed by intracellular staining of IL-2, IFN-γ (top), TNF-α and CD107a (bottom). **(D)** IFN-γ concentration in the supernatant of overnight, 1:1 ratio co-culture of AFP TCR T cells with HepG2 target cells or control Huh7 cells measured by ELISA. Data represents the mean + s.d. of quadruplicate co-culture samples. **(E)** Cytolytic capacity of AFP TCR T cells toward HepG2 target cells over a 4-day co-culture. Data represents the mean of triplicate samples derived from RTCA instrument, as compared to target only wells. (E:T): effect to target ratio. Colors are represented as in **(D)**. **(F)** Time to eradicate 50% of target cells (KT50) of different AFP TCR T cells at the indicated effect to target (E:T) ratio. Colors are represented as in **(D)**. T cells transduced with TCR 8 and TCR 11 did not eradicate 50% of the target by the end of the analysis at E:T = 0.25:1, thus were not plotted. Data shown in b-f is representative of at least 3 independent experiments on T cells isolated from 3 healthy donors. UT, untransduced T cells.

We have previously reported the identification of 3 TCRs from HLA-A2 transgenic AAD mice after immunization with human AFP_158−166_ peptide (19). Four additional TCRs were identified from newly sequenced hybridomas, following the same method described previously (19). In order to identify the optimal AFP TCRs based on potency toward their targets, we first codon-optimized and cloned the 7 AFP TCRs (19) into a lenti-viral backbone and enforced their expression in human T cells. Eight days after transduction, the relative T cell expansion and phenotype of 7 AFP TCR-transduced cells are comparable (Figure S1). Next we evaluated the expression of TCR β chain as well as the pairing of TCR α and β chains by measuring the surface expression levels of TCR β chain and AFP tetramer binding capacities. The expression level of TCR β chains is comparable, ranging from 80 to 90% for these 7 TCRs. Interestingly, the tetramer binding capacity of these TCRs is highly variable, although the CDR3 regions of these TCRs are quite similar at the amino acid level especially when compared in pairs (Figure 1A). TCR 3 demonstrated the highest tetramer binding capacity reaching almost 60%, followed by TCR 1, 2, and 6, reaching 40%, while TCR 8, 10, and 11 were much lower in the range of 7–26% (Figure 1B). We reasoned that the different tetramer binding activity is very likely due to the pairing difference between TCR α and β chain given the comparable expression level of 7 TCR β chains and the assumption that the expression of TCR α chain is similar since the codon-optimized α chain is cloned upstream of TCR β chain.

To evaluate the cytokine secretion capacity of these AFP TCRs, we co-cultured the TCR expressing cells with HCC cell line HepG2, which displays high AFP expression in a HLA-A2 setting (26). A significant fraction of T cells redirected with TCR 1, 2, and 3 were capable of producing multiple cytokines including IL-2, IFN-γ, TNF-α, as well as degranulation factor CD107a. Nevertheless, TCR 8 and 11 expressing T cells produced very few cytokines and TCR 6 and 10 expressing T cells displayed intermediate cytokine producing capacity (Figure 1C). This observation is consistent with their respective tetramer binding capacities, except for TCR 6 expressing T cells, which produced less cytokines compared to TCR 1 and 2 redirected T cells, even though their tetramer binding capacities were close, suggesting that the cytokine production capacity is not solely dependent on the ability to bind target. Consistently, the IFN-γ secretion capacity of different TCRs aligned well with their intracellular cytokine production capacities, i.e., T cells expressing TCR 1, 2, and 3 secreted the highest amount of IFN-γ (Figure 1D).

We next examined the cytolytic activity of these TCR expressing cells upon encountering their targets. Again, T cells expressing TCR 1, 2, and 3 displayed the highest cytotoxicity, killing almost 95% target HepG2 cells after co-culturing for 48 h (Figure 1E) and taking virtually the same time to kill 50% of the targets at the effector to target ratio of 0.25:1 and 0.5:1, surpassing the other 4 TCRs (Figure 1F). Taken together, TCR 1, 2, and 3 demonstrated the best TCR expression and pairing profile, strong cytokine production capacity, and most potent target killing efficacy. These three TCRs were therefore selected for the next step of safety profile screening.

### Selection of AFP TCR 2 Based on Its Balance of Potency and Specificity

**Figure 2:**
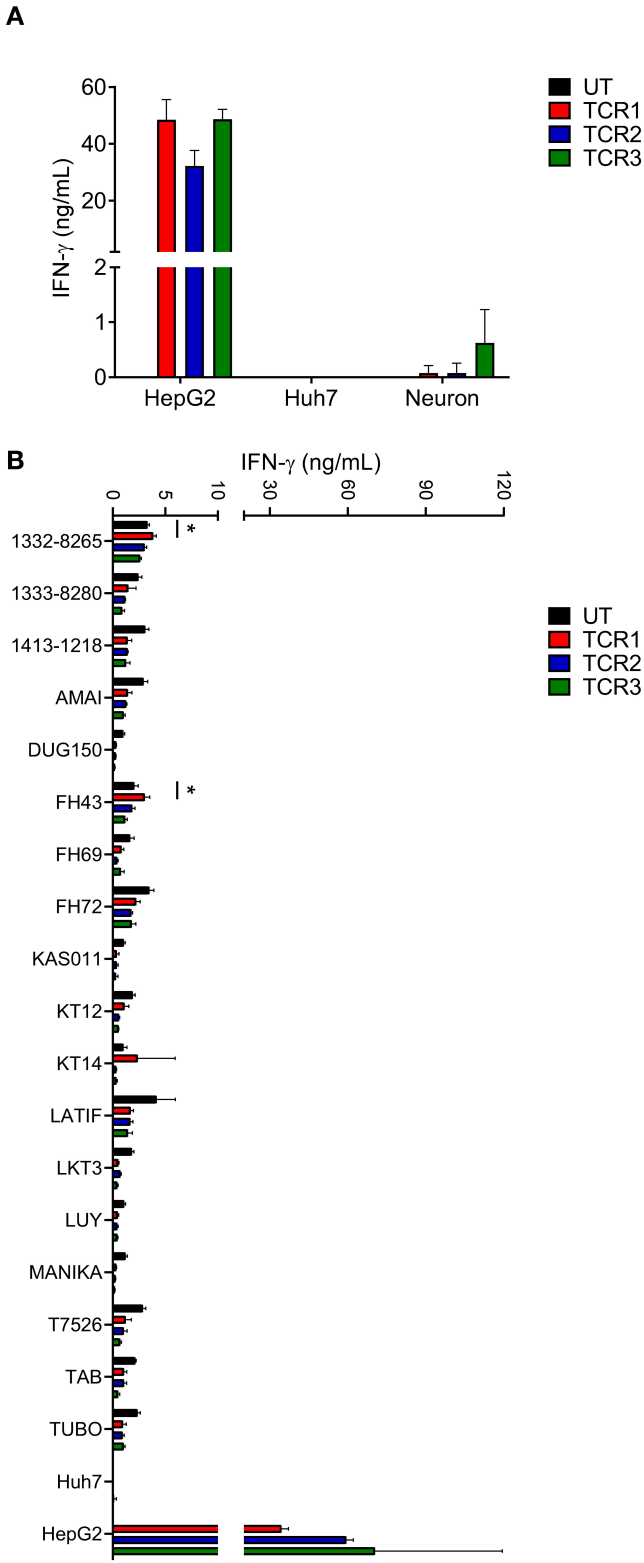
AFP TCR 2 shows lower than background activity toward primary neurons and no alloreactivity compared to TCR 1 and 3. **(A)** IFN-γ concentration in the supernatant of overnight, 1:1 ratio co-culture of the indicated AFP TCR T cells with iCell derived neurons measured by ELISA. Data represents the mean + s.d. of the triplicate co-cultures and is representative of T cells prepared from 2 healthy donors. **(B)** IFN-γ concentration in the supernatant of overnight, 1:1 ratio co-culture of the indicated AFP TCR T cells with a panel of Epstein-Barr virus transformed B cell lines expressing various HLA alleles measured by ELISA. Data represents the mean + s.d. of quadruplicate co-cultures and is representative of T cells prepared from 2 healthy donors. HepG2 and Huh7 cells were included as positive and negative controls, respectively, in both **(A,B)**. *P < 0.05 (unpaired two-tailed Student's *t*-test).

AFP protein shows a specific expression pattern in HCC that rarely appears in human adult tissue (13, 15). However, to more rigorously evaluate potential “on-target, off-tumor” toxicity for AFP TCRs, we examined the ability of AFP TCR engineered T cells to react with a range of primary cells, especially those from essential human organs. First we tested the reactivity of AFP TCR transduced cells to HLA-A^*^02:01^+^ iCell derived neurons. T cells transduced with TCR 1 and 2 showed minimal reactivity against iCell neurons but the reactivity of TCR 3 transduced cells was relatively higher in one of the two donor cells tested (Figure 2A and the data of the other donor is not shown), suggesting that TCR 3 is less specific compared to TCR 1 and 2.

Simultaneously, we tested the reactivity of TCR 1, 2, and 3 against a panel of EBV–transformed B cell lines designed to evaluate alloreactivity (15, 22). We did observe low level of IFN-γ release from untransduced (UT) control T cells after co-culture with these B cell lines, most likely due to the recognition of EBV antigen by the endogenous TCRs. Therefore, only increases in IFN-γ release above such background would be considered as alloreactive. To this end, TCR 1 engineered T cells did show higher activity against cell line 1332-8265, FH43, and KT14 compared to other T cell products including UT, TCR 2 and 3 engineered T cells (Figure 2B), suggesting that the relative alloreactivity of TCR 1 is higher than the other two TCRs.

Taken together, we decided to focus on TCR 2 to further test the toxicity of this TCR as our leading clinical candidate based on its optimal balance of activity and specificity relative to TCR 1 and 3. Furthermore, TCR 2 also displayed strong anti-tumor activity in an NSG mouse model (19), consolidating the choice of this TCR for further safety profile testing.

### No Off-Target Recognition Was Detected for TCR 2 Engineered Cells Toward a Panel of HLA-A^*^02:01^+^ Primary Cells

**Figure 3:**
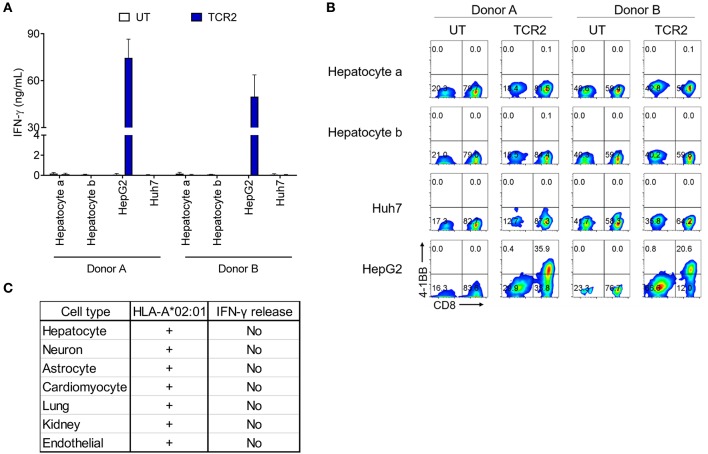
AFP TCR 2 displays no reactivity toward a variety of HLA-A*02:01^+^ primary cells. **(A)** IFN-γ concentration in the supernatant of overnight, 1:1 ratio co-culture of UT or T cells expressing AFP TCR 2 with primary hepatocytes measured by ELISA. T cells were prepared from 2 healthy donors and human primary hepatocytes were obtained from 2 adult HLA-A*02:01^+^ donors. Data represents the mean + s.d. of quadruplicate co-cultures. **(B)** 4-1BB upregulation by T cells after co-culture as described in a. Cells were further analyzed by FACS for surface expression of 4-1BB on total CD3^+^ untransduced T cells or AFP TCR 2 transduced T cells. HepG2 and Huh7 cells were included as positive and negative controls, respectively, in both **(A,B)**. **(C)** Summary of reactivity from co-culture experiment of T cells expressing AFP TCR 2 with primary hepatocytes, primary lung and kidney epithelial cells, and iCell derived primary cells including neurons, astrocytes, cardiomyocyte, and endothelial cells.

To exclude any potential cross-reactivity of AFP TCR 2 engineered T cells against essential human tissues, we further tested the reactivity of TCR 2 expressing T cells toward a panel of HLA-A^*^02:01^+^ primary cells derived from different human tissues. First we co-cultured AFP TCR 2 engineered T cells with primary hepatocytes from various donors and resources and measured the IFN-γ release as an indicator of reactivity. Again, TCR 2 engineered T cells released high levels of IFN-γ toward their target HepG2 cells, but only background levels of IFN-γ were detected when co-culturing with primary hepatocytes (Figure 3A). Similarly, no significant amount of cytokines or effector molecules were detected from the co-culture supernatant including IL-2, IL-4, IL-6, IL-10, IL-17a, TNF-α, sFas, sFasL, Granzyme A, Granzyme B, Perforin, and Granulysin (Figure S2). This suggests that there is no cross-reactivity of TCR2 engineered T cells with primary hepatocytes, further reassuring that AFP is a safe target for HCC. To further test the potential reactivity on a per cell basis, we checked the 4-1BB level in TCR 2 engineered T cells co-cultured with primary hepatocytes. No up-regulation of 4-1BB was detected in TCR 2 engineered cells co-cultured with hepatocytes while a significant fraction of 4-1BB was upregulated when co-cultured with HepG2 cells, demonstrating that TCR 2 engineered T cells do not recognize primary HLA-A^*^02:01^+^ hepatocytes (Figure 3B).

We therefore performed serials of assays including IFN-γ release and 4-1BB upregulation by TCR 2 engineered T cells co-cultured with a number of HLA-A^*^02:01^+^ primary or iCell derived cells including astrocytes, cardiomyocytes, lung, kidney, and endothelial cells. Thus far, there was no above background cross-reactivity detected (Figure 3C and Figure S3). Taken together, we conclude that TCR 2 engineered T cells demonstrate no above-background cross-reactivity toward the HLA-A^*^02:01^+^ primary cells tested.

### TCR 2 Engineered T Cells Showed Minimal Alloreactivity

**Figure 4:**
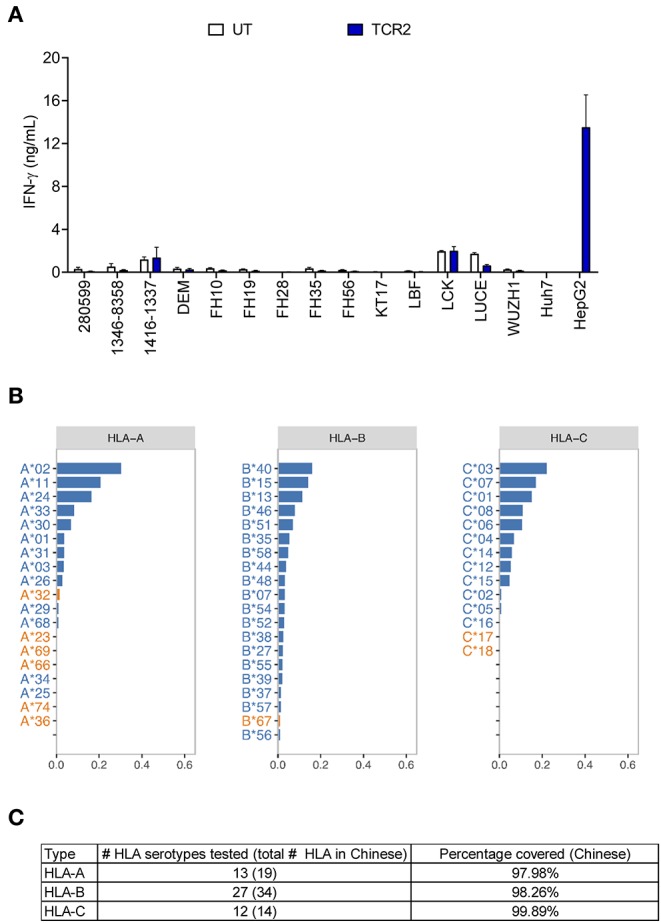
AFP TCR 2 shows no alloreactivity. **(A)** IFN-γ concentration in the supernatant of overnight, 1:1 ratio co-culture of UT or AFP TCR 2 transduced T cells with a panel of 14 Epstein-Barr virus transformed B cell lines expressing various HLA alleles measured by ELISA. Data represents the mean + s.d. of quadruplicate co-cultures and is representative of 3 healthy donors. A total of 38 transformed B cell lines were tested. **(B)** Frequencies of class I HLA serotypes in Chinese Han population. Serotypes were sorted by frequency for each subclass from high to low; those covered by the 38 transformed B cell lines in our test are shown in blue while those not covered are shown in orange. For HLA-B, only the top 20 serotypes are displayed. **(C)** Summary of the number of class I HLA serotypes covered in this study and corresponding cumulative percentage in Chinese Han population.

Next we performed a comprehensive screening of the alloreactivity of TCR 2 engineered T cells using a large panel of EBV-transformed B cell lines, which has been widely used as a surrogate for the antigen presenting cells with different HLA types (15, 22). Due to the large number of HCC cases and unmet medical need in the Chinese population, we first chose EBV-transformed B cell lines covering a significant percentage of HLA serotypes in Chinese population (27). In addition to the previous 14 EBV-transformed lines tested (Figure 2B), we examined another set of EBV-transformed B cell lines (Figure 4A) and later expanded to a total of 38 lines (Table S1), covering more than 98% of the HLA class I serotype of Chinese population (Figures 4B,C). There was generally no above-background level of IFN-γ detected in the allo-lines tested (Figure 4A and the data of other 10 cell lines is not shown). However, in the co-culture supernatant from cell line 1332-8265 and FH43, we found that the IFN-γ release was slightly higher than UT, although this observation was donor dependent (Figure S4A). All the HLA class I alleles expressed by these two cell lines are also found in other non-reactive cell lines. However, they share a common HLA class II allele DPB1^*^11:01, which is unique to these two B cell lines and absent from the rest in the same experiment. To test whether there is alloreactivity of TCR 2 against DPB1^*^11:01^+^ cells, in an independent experiment we measured the 4-1BB upregulation in TCR 2 engineered T cells against 1332-8265 and FH43, together with an additional DPB1^*^11:01^+^ line FH5, and other DPB1^*^11:01 negative controls. Again, 4-1BB was highly upregulated when TCR 2 engineered CD8^+^ T cells encountered the target cell HepG2. We also detected marginal upregulation of 4-1BB in the CD4^+^ fraction of TCR 2 engineered T cells, when they were co-cultured with all three DPB1^*^11:01^+^ cell lines from our collection of 38 lines. We considered it marginal because the highest percentage of upregulation was only about 1.8%, compared to the background 0.4% in its UT counterpart. Interestingly, we did not observe upregulation in TCR 2 engineered CD8^+^ T cells (Figure S4B), suggesting that the reactivity to DPB1^*^11:01 is restricted to CD4^+^ T cells transduced with TCR 2. To further examine whether CD4^+^ T cells are the only resource inducing the alloreactivity, we tested the alloreactivity to DPB1^*^11:01 cell lines with AFP TCR 2 engineered pan T cells including both CD4^+^ and CD8^+^ T subsets and AFP TCR 2 engineered CD8^+^ only T cells from the same donor. Consistent to our hypothesis, the increased IFN-γ release compared to UT from pan T cells diminished in CD8^+^ T cell only population (Figure S4C), suggesting that the minimal reactivity to DPB1^*^11:01 allele is associated with TCR 2 transduced CD4^+^ T cells. In other words, TCR 2 may show minimal alloreactivity to DPB1^*^11:01 allele.

In total we have tested 38 EBV-transformed B cell lines covering 52 HLA serotypes, which represent more than 98% of HLA-A, B, and C in the Chinese population, accounting for almost half of the HCC patients worldwide (Figures 4B,C). These selected B cell lines also cover a significant percent of other ethnic groups including European Caucasian, African American, Hispanic, Mexican/Chicano, South Asian Indian based on data from the Allele Frequency Net Database (28) (Table S2). This shows the eligibility of a significantly greater patient population for this AFP TCR worldwide.

### AFP TCR 2 Is Unlikely to Cross-React With Other Proteins in the Human Genome

**Figure 5:**
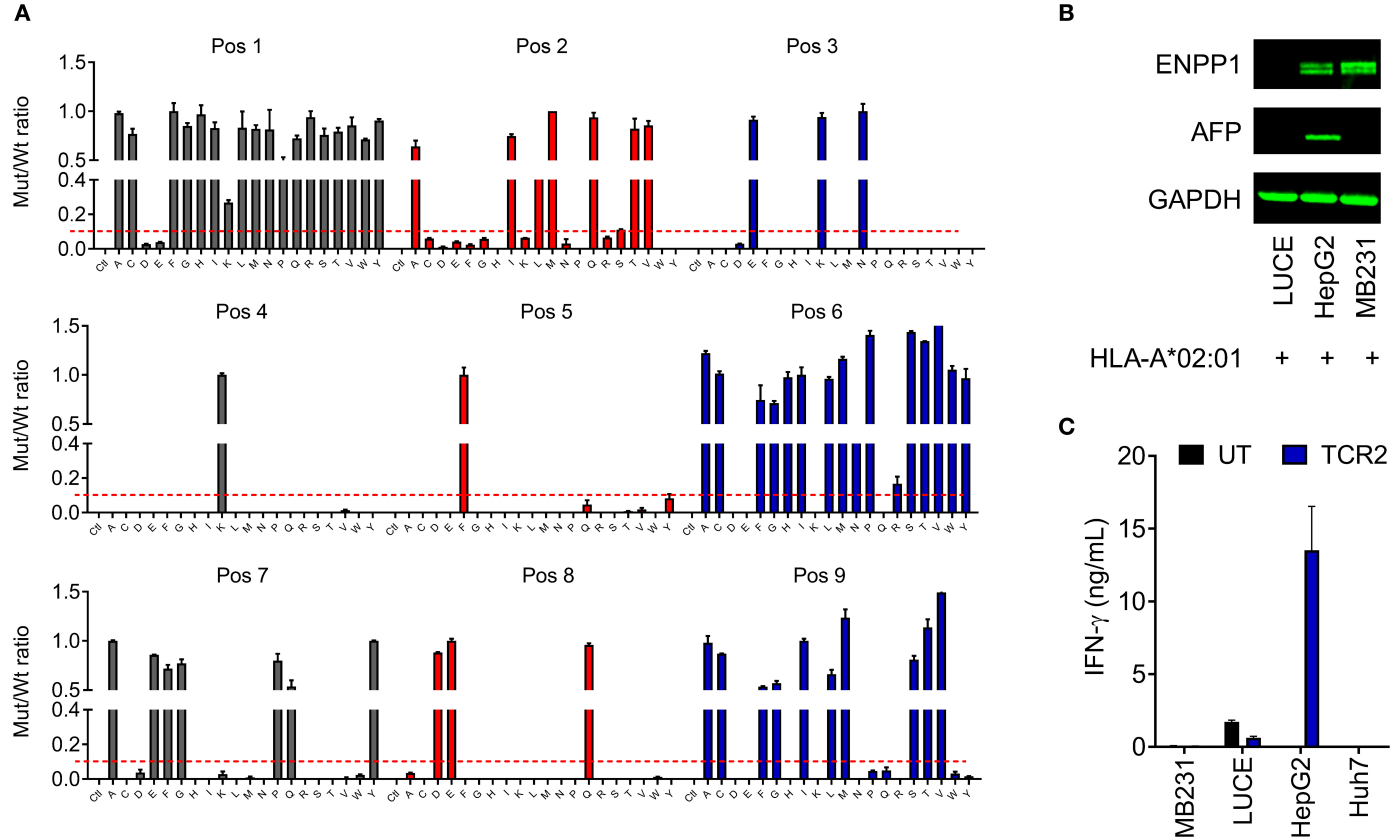
AFP TCR 2 is unlikely to cross-react with other proteins in the human genome. **(A)** Graphic presentation of X-scan results showing the replaceability of each amino acid of AFP_158−166_ epitope. Peptides with replacement of each amino acid residue with every other 19 amino acids were used to activate the TCR 2 transduced T cells. The amount of IFN-γ produced by AFP TCR 2 transduced T cells after stimulation with mutated epitope (mut) was compared to that from wild type AFP_158−166_ peptide (wt) and the ratios are presented. Dashed line indicates the cut-off for tolerance. Data represents the mean + s.d. of duplicate co-cultures and is representative of 3 healthy donors. **(B)** ENPP1 and AFP expression in the indicated cell lines tested by Western blot. GAPDH was used as loading control. **(C)** IFN-γ concentrations in the supernatant of overnight, 1:1 ratio co-culture of UT or AFP TCR 2 transduced T cells with the indicated cell lines measured by ELISA. Data represents the mean + s.d. of quadruplicate co-cultures and is representative of 3 healthy donors.

In order to detect whether there is any cross-reactivity of AFP TCRs to any other proteins in the human genome, an X-scan screen was performed to exclude the possibilities [accompanied study, (24)]. Basically we identified the key irreplaceable amino acids of AFP_158−166_ peptide for TCR 2 recognition (Figure 5A). In other words, any amino acid replacement that induces more than 90% activity loss is considered as irreplaceable. Using this criteria, we identified an AFP TCR 2 binding motif defining the essential positions critical for AFP TCR 2 activity (Figure 5A).

We next performed a BLAST search of the UniProtKB protein database with the identified key motif for AFP TCR 2. Further analysis and testing identified peptide YLNKYLGDV as the only candidate that is able to activate TCR 2 transduced T cells *in vitro* [for details see the accompanied study, (24)].

YLNKYLGDV could potentially be processed from transmembrane glycoprotein ectonucleotide pyrophosphatase phosphodiesterase 1 (ENPP1) protein, a key player involved in inhibiting insulin receptor signaling and the development of insulin resistance (29). We next set to test whether ENPP1 expressing cells can activate TCR 2 transduced T cells. It is found that ENPP1 is highly expressed in MDA-MB-231, a HLA-A^*^02:01^+^ cell line, according to the Broad Institute Cancer Cell Line Encyclopedia^1^. We therefore evaluated the ENPP1 level in MDA-MB-231 cells by Western blot. Consistent with the reported mRNA expression in the database, ENPP1 protein was expressed in MDA-MB-231 cells, similar to the level in HepG2 cells (Figure 5B). Interestingly, ENPP1 protein was also detected in primary hepatocytes [accompanied study, (24)], however AFP was only detected in HepG2 cells (Figure 5B). We therefore performed a co-culture assay to test the reactivity of TCR 2 transduced T cells toward different cell lines. Again, high IFN-γ release was detected from the co-culture supernatant of HepG2 cells, but there was virtually no IFN-γ release from the co-culture supernatant of MDA-MB-231 (Figure 5C). Previously, we also showed that there is no IFN-γ or other cytokine release as well as T cell activation when TCR 2 expressing cells were co-cultured with primary hepatocytes (Figures 3A,B and Figure S2). Taken together, it is clear that TCR 2 transduced T cells do not recognize HLA-A^*^02:01 restricted, ENPP1 expressing cells but only recognize AFP expressing targets. Because ENPP1 is the only candidate that may be recognized by AFP TCR 2 in X-scan screening, we can conclude that there is virtually no cross-reactivity of TCR 2 transduced T cells to other antigens in the human genome under physiological condition.

## Discussion

In this article, we described a pipeline for systematically selecting a clinical lead AFP TCR for translational purposes. We first screened our TCR candidates based on their reactivity toward the target *in vitro*. Once we shrunk the pool of candidates by selecting the most potent ones, we further tested their cross-reactivity toward primary cells, EBV-transformed B cell lines covering a wide range of HLA class I, and also reactivity toward other potential targets in the human genome. Based on the balance of all the above factors, we selected TCR 2 to start our clinical trial (NCT03971747).

Our TCR pool is generated from natural occurring TCRs after immunizing AAD mice with AFP_158−166_ peptide (19). Interestingly, these TCRs displayed different TCR α and β chain pairing capability and also varied activities with a few amino acid differences in the CDR3 region. We found that the CDR3 region of our TCR β chains is quite conserved, with only 2–3 amino acid differences (Figure 1A), which is consistent with the findings that different TCR clonotypes specific for the same antigen often utilize the same Vβ gene segment (30, 31). However, we observed significant differences among the activity of these TCRs. For example, TCR 3 and TCR 8 only vary by 3 amino acids in the CDR3 region: 1 in the β chain and 2 in the α chain. However, the pairing capacity of their α and β chains is quite different and the reactivity against targets is even more different in the *in vitro* assays (Figures 1B–F), suggesting that only a few amino acid changes in CDR3 region can cause substantial difference in TCR activities. Because all of these TCRs are naturally occurring, the information embedded in their CDR3 region together with their corresponding activity can provide a reference for gene engineering strategies aimed to improve TCR affinity and potency.

For effective adoptive cell therapy, one would like to choose a TCR with optimal affinity to allow the TCR engineered T cells to efficiently eradicate their targets. However, TCRs with higher activity are not always the best clinical option as they may pose a safety issue. It is established that TCRs of high affinity within the natural range are more efficacious but also prone to be associated with less specificity (32). In our case, TCR 1 and 3 most times outperformed TCR 2 (we repeated the same result many times besides the data shown in Figures 1D–F and Figure 2) in IFN-γ release. But the increase of activity, again, is associated with a higher level of reactivity toward primary cells and also alloreactivity. Furthermore, TCR 2 also displayed potent anti-tumor activity in an NSG mouse model (19). Taken together, we chose TCR 2 as our clinical lead candidate even though the anti-tumor activity is comparable or even less potent compared to TCR 1 and 3 *in vitro*.

All of our TCRs are not gene engineered so the chance of non-specificity induced by gene engineering is relatively low. However, we think it is still critical and necessary to perform safety studies to select the best candidate for clinical use. Unfortunately, there are no optimal animal models to test the cross-reactivity of TCRs to human tissues due to the absence of human antigen and HLA expression and processing in these models. Therefore, carefully designed *in vitro* assays become critical. We, again, confirmed that the combination of using primary cells, EBV-transformed cell lines with different HLA alleles, and X-scan is a valid platform for evaluating TCR safety profile, which is supported by numerous retrospective and prospective studies (15, 22, 33, 34). Although the actual safety of a TCR needs to be eventually evaluated by carefully designed phase I dose-escalation safety trials, which are now performed by almost all T cell-based therapy trials, it is worth the effort to perform an *in vitro* preclinical safety analysis using the above methodologies considering the low cost and the quick turnaround of these *in vitro* assays compared to clinical trials.

Considering the urgent unmet medical need for treating HCC in China and worldwide, adoptive cell transfer appears to be a promising method to effectively treat this malignancy. Our study provides a basis for advancing a TCR targeting HCC into the clinic based on the balance of activity and safety. The extensive panel of selected EBV-transformed cell lines in our assay covers majority of HLA serotypes, offering a selection criterion for patient recruitment across a wide range of ethnic groups worldwide. Furthermore, our study also provides a reference for generating different recruitment criteria for patients from different ethnic backgrounds. For example, we did not exclude DPB1^*^11:01 in our first-in-human trial in Han Chinese due to the following reasons: First, the percentage of DPB1^*^11:01 in Han Chinese, which accounts for 92% of Chinese population, is just 0.03%. Considering that all the patients we plan to recruit must be A^*^02:01^+^, which is around 14%, the chance of encountering an A^*^02:01^+^ DPB1^*^11:01^+^ patient is extremely low. Second, the alloreactivity of TCR 2 toward DPB1^*^11:01 positive cells is relatively marginal, only around 2% of the amount from positive control HepG2 cells, and is donor dependent. Therefore, we believe it is relatively safe to move TCR 2 further into clinical trial without excluding DPB1^*^11:01^+^ patients in Han Chinese. However, it will be necessary to exclude DPB1^*^11:01 if the patient pool includes a higher percentage of this allele. Currently an early phase clinical trial using T cells transduced with TCR 2 to treat HCC patients (NCT03971747) has been initiated in China. We hope the safety and efficacy data obtained from this trial will provide a solid foundation for its application worldwide.

In conclusion, we have selected a TCR with a balance of affinity, function, and safety profile, bearing properties that are expected to allow AFP TCR redirected T cells to specifically differentiate between AFP levels on tumor and normal tissues. The safety and efficacy signals obtained from the clinical trial (NCT03971747) with this TCR will provide a solid foundation for its application worldwide.

## Data Availability Statement

The raw data supporting the conclusions of this article will be made available by the authors, without undue reservation, to any qualified researcher.

## Author Contributions

YJ, XL, LC, and YH designed and interpreted the study. XL, HC, LC, W-CY, and MP performed the experiments. XL, YJ, HC, SZ, JH, XY, and YY analyzed the data. WZ performed bioinformatics analysis. YJ and XL wrote the manuscript. All authors read and approved the final manuscript.

### Conflict of Interest

XL, HC, W-CY, MP, SZ, JH, XY, YY, and YJ are employees of Cellular Biomedicine Group Inc. YH is a consultant of Cellular Biomedicine Group Inc. WZ is employed by CodexSage LLC. The remaining authors declare that this study received funding from Cellular Biomedicine Group Inc. The funder had the following involvement with the study: the study design, collection, analysis, interpretation of data, the writing of this article and the decision to submit it for publication.

## Footnotes

^1^https://portals.broadinstitute.org/ccle

## Abbreviations

HCC: hepatocellular carcinoma
AFP: alpha-fetoprotein
UT: untransduced
EBV: Epstein-Barr virus
ELISA: enzyme-linked immunosorbent assay
IFN: interferon
PBMC: peripheral blood mononuclear cells
TCR: T cell receptor
BLAST: Basic Local Alignment Search Tool.

## References

[1] Bray F Ferlay J Soerjomataram I Siegel RL Torre LA Jemal A. Global cancer statistics 2018: GLOBOCAN estimates of incidence and mortality worldwide for 36 cancers in 185 countries. CA Cancer J Clin. (2018) 68:394–424. 10.3322/caac.21492 30207593

[2] Chen W Zheng R Baade PD Zhang S Zeng H Bray F. Cancer statistics in China, 2015. CA Cancer J Clin. (2016) 66:115–32. 10.3322/caac.21338 26808342

[3] Wege H Li J Ittrich H. Treatment lines in hepatocellular carcinoma. Visc Med. (2019) 35:266–72. 10.1159/000501749 31602390 PMC6738173

[4] Yao W He JC Yang Y Wang JM Qian YW Yang T. The prognostic value of tumor-infiltrating lymphocytes in hepatocellular carcinoma: a systematic review and meta-analysis. Sci Rep. (2017) 7:7525. 10.1038/s41598-017-08128-1 28790445 PMC5548736

[5] Ding W Xu X Qian Y Xue W Wang Y Du J. Prognostic value of tumor-infiltrating lymphocytes in hepatocellular carcinoma: a meta-analysis. Medicine. (2018) 97:e13301. 10.1097/MD.0000000000013301 30557978 PMC6320107

[6] Shen Y Xia M Zhang J Xu L Yang J Chen A. IRF-1 and p65 mediate upregulation of constitutive HLA-A antigen expression by hepatocellular carcinoma cells. Mol Immunol. (2009) 46:2045–53. 10.1016/j.molimm.2009.03.001 19428110 PMC3426235

[7] Rosenberg SA Restifo NP. Adoptive cell transfer as personalized immunotherapy for human cancer. Science. (2015) 348:62–8. 10.1126/science.aaa4967 25838374 PMC6295668

[8] June CH O'Connor RS Kawalekar OU Ghassemi S Milone MC. CAR T cell immunotherapy for human cancer. Science. (2018) 359:1361–5. 10.1126/science.aar6711 29567707

[9] Majzner RG Mackall CL. Clinical lessons learned from the first leg of the CAR T cell journey. Nat Med. (2019) 25:1341–55. 10.1038/s41591-019-0564-6 31501612

[10] Ji Y Hocker JD Gattinoni L. Enhancing adoptive T cell immunotherapy with microRNA therapeutics. Semin Immunol. (2016) 28:45–53. 10.1016/j.smim.2015.11.006 26710685 PMC4862908

[11] D'Ippolito E Schober K Nauerth M Busch DH. T cell engineering for adoptive T cell therapy: safety and receptor avidity. Cancer Immunol Immunother. (2019) 68:1701–12. 10.1007/s00262-019-02395-9 31542797 PMC11028346

[12] Wolf B Zimmermann S Arber C Irving M Trueb L Coukos G. Safety and tolerability of adoptive cell therapy in cancer. Drug Saf. (2019) 42:315–34. 10.1007/s40264-018-0779-3 30649750

[13] Galle PR Foerster F Kudo M Chan SL Llovet JM Qin S. Biology and significance of alpha-fetoprotein in hepatocellular carcinoma. Liver Int. (2019) 39:2214. 10.1111/liv.14223 31436873

[14] Sun L Guo H Jiang R Lu L Liu T He X. Engineered cytotoxic T lymphocytes with AFP-specific TCR gene for adoptive immunotherapy in hepatocellular carcinoma. Tumour Biol. (2016) 37:799–806. 10.1007/s13277-015-3845-9 26250457

[15] Docta RY Ferronha T Sanderson JP Weissensteiner T Pope GR Bennett AD. Tuning T-cell receptor affinity to optimize clinical risk-benefit when targeting alpha-fetoprotein-positive liver cancer. Hepatology. (2019) 69:2061–75. 10.1002/hep.30477 30561769 PMC6593660

[16] Liu H Xu Y Xiang J Long L Green S Yang Z. Targeting alpha-fetoprotein. (AFP)-MHC complex with CAR T-cell therapy for liver cancer. Clin Cancer Res. (2017) 23:478–88. 10.1158/1078-0432.CCR-16-1203 27535982

[17] Lipika Goyal MF Meyer T Lynn G Jordi B El-Khoueiry A Hausner P Abstract 3183: initial safety of AFP SPEAR T-cells in patients with advanced hepatocellular carcinoma. Cancer Res. (2019) 79:3183 10.1158/1538-7445.AM2019-3183

[18] Eureka Therapeutics Achieves Regression Of Metastatic Liver Cancer Using Et140202 T-Cell Therapy 2019. Available online at: https://www.eurekatherapeutics.com/media/press-releases/090518/ (accessed April 1, 2020).

[19] Zhu W Peng Y Wang L Hong Y Jiang X Li Q. Identification of alpha-fetoprotein-specific T-cell receptors for hepatocellular carcinoma immunotherapy. Hepatology. (2018) 68:574–89. 10.1002/hep.29844 29443377 PMC7368991

[20] Johnson LA Morgan RA Dudley ME Cassard L Yang JC Hughes MS. Gene therapy with human and mouse T-cell receptors mediates cancer regression and targets normal tissues expressing cognate antigen. Blood. (2009) 114:535–46. 10.1182/blood-2009-03-211714 19451549 PMC2929689

[21] Parkhurst MR Yang JC Langan RC Dudley ME Nathan DA Feldman SA. T cells targeting carcinoembryonic antigen can mediate regression of metastatic colorectal cancer but induce severe transient colitis. Mol Ther. (2011) 19:620–6. 10.1038/mt.2010.272 21157437 PMC3048186

[22] Cameron BJ Gerry AB Dukes J Harper JV Kannan V Bianchi FC. Identification of a Titin-derived HLA-A1-presented peptide as a cross-reactive target for engineered MAGE A3-directed T cells. Sci Transl Med. (2013) 5:197ra103. 10.1126/scitranslmed.3006034 23926201 PMC6002776

[23] Morgan RA Chinnasamy N Abate-Daga D Gros A Robbins PF Zheng Z. Cancer regression and neurological toxicity following anti-MAGE-A3 TCR gene therapy. J Immunother. (2013) 36:133–51. 10.1097/CJI.0b013e3182829903 23377668 PMC3581823

[24] Cai L Caraballo Galva LD Peng Y Luo X Zhu W Yao Y Preclinical Studies of the Off-Target Reactivity of AFP158-Specific TCR Engineered T Cells. Front Immunol. (2020) 11:607 10.3389/fimmu.2020.00607 PMC7196607 32395117

[25] Gatz SA Pohla H Schendel DJ. A PCR-SSP method to specifically select HLA-A^*^0201 individuals for immunotherapeutic studies. Tissue Antigens. (2000) 55:532–47. 10.1034/j.1399-0039.2000.550604.x 10902609

[26] Kawai HF Kaneko S Honda M Shirota Y Kobayashi K. alpha-fetoprotein-producing hepatoma cell lines share common expression profiles of genes in various categories demonstrated by cDNA microarray analysis. Hepatology. (2001) 33:676–91. 10.1053/jhep.2001.22500 11230749

[27] Zhou F Cao H Zuo X Zhang T Zhang X Liu X. Deep sequencing of the MHC region in the Chinese population contributes to studies of complex disease. Nat Genet. (2016) 48:740–6. 10.1038/ng.3576 27213287

[28] Gonzalez-Galarza FF Takeshita LY Santos EJ Kempson F Maia MH da Silva AL. Allele frequency net 2015 update: new features for HLA epitopes, KIR and disease and HLA adverse drug reaction associations. Nucleic Acids Res. (2015) 43(Database issue):D784–8. 10.1093/nar/gku1166 25414323 PMC4383964

[29] Bacci S De Cosmo S Prudente S Trischitta V. ENPP1 gene, insulin resistance and related clinical outcomes. Curr Opin Clin Nutr Metab Care. (2007) 10:403–9. 10.1097/MCO.0b013e3281e386c9 17563456

[30] Price DA Asher TE Wilson NA Nason MC Brenchley JM Metzler IS. Public clonotype usage identifies protective Gag-specific CD8+ T cell responses in SIV infection. J Exp Med. (2009) 206:923–36. 10.1084/jem.20081127 19349463 PMC2715115

[31] Valkenburg SA Josephs TM Clemens EB Grant EJ Nguyen TH Wang GC. Molecular basis for universal HLA-A^*^0201-restricted CD8+ T-cell immunity against influenza viruses. Proc Natl Acad Sci USA. (2016) 113:4440–5. 10.1073/pnas.1603106113 27036003 PMC4843436

[32] Irving M Zoete V Hebeisen M Schmid D Baumgartner P Guillaume P. Interplay between T cell receptor binding kinetics and the level of cognate peptide presented by major histocompatibility complexes governs CD8+ T cell responsiveness. J Biol Chem. (2012) 287:23068–78. 10.1074/jbc.M112.357673 22549784 PMC3391157

[33] Heemskerk MH de Paus RA Lurvink EG Koning F Mulder A Willemze R. Dual HLA class I and class II restricted recognition of alloreactive T lymphocytes mediated by a single T cell receptor complex. Proc Natl Acad Sci USA. (2001) 98:6806–11. 10.1073/pnas.111162298 11381117 PMC34434

[34] Obenaus M Leitao C Leisegang M Chen X Gavvovidis I van der Bruggen P. Identification of human T-cell receptors with optimal affinity to cancer antigens using antigen-negative humanized mice. Nat Biotechnol. (2015) 33:402–7. 10.1038/nbt.3147 25774714

