# Supplementary material for: Selection of a Clinical Lead TCR Targeting Alpha-Fetoprotein-Positive Liver Cancer Based on a Balance of Risk and Benefit
Source: Front Immunol. 2020 Apr 27;11:623. doi: 10.3389/fimmu.2020.00623 (PMC7203609; doi:10.3389/fimmu.2020.00623)
Supplement: Supplementary file 1 [file Data_Sheet_1.PDF]

Supplementary Material
Figure S1

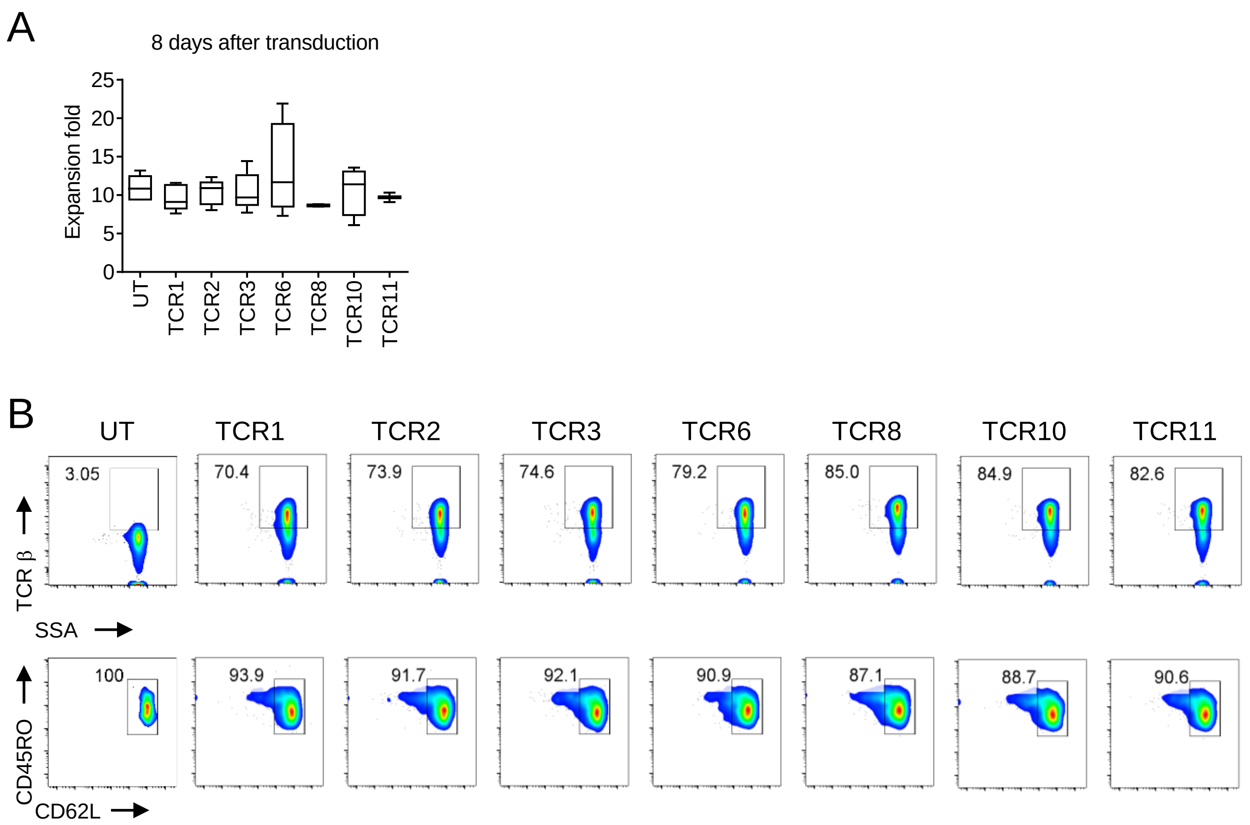

Figure S1. T cells transduced with seven indicated AFP TCRs demonstrate comparable T cell expansion and phenotype. (**A**) Relative T cell expansion eight days after transducing of indicated AFP TCRs. (**B**) FACS analysis of the surface expression of mouse TCR β chain, CD45RO, and CD62L eight days after transducing of indicated AFP TCRs. The percentage of TCR β^+ (top) or $CD62L^+$ $CD45RO^+$ Tcm (bottom, gated on TCR β^+ population) cells are indicated. Data is representative of at least two independent experiments in which T cells were prepared from 2 healthy donors.

Figure S2

A

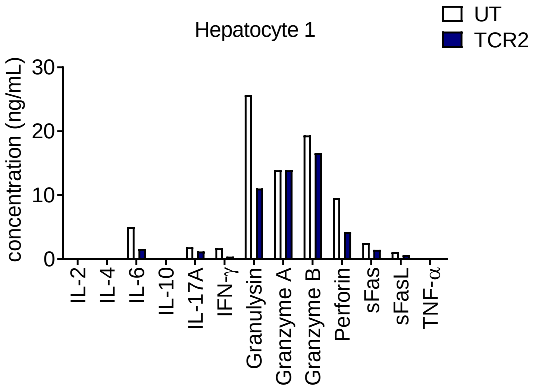

B

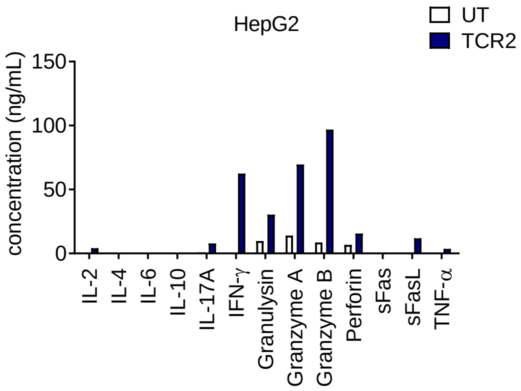

Figure S2. AFP TCR 2 shows no reactivity towards HLA-A*02:01^{+} primary hepatocytes. (**A**, **B**) Concentration of the indicated cytokines in the co-cultured supernatant of T cells transduced with AFP TCR 2 or the untransduced control (UT) with HLA-A*02:01^{+} primary hepatocytes (**A**) or HepG2 target cells (**B**). The concentration of the indicated cytokines in the co-cultured supernatant was determined by a bead-based immunoassay run on a FACS instrument. Data is shown as geometric mean concentrations of each cytokine tested. Data is representative of two independent experiments in which T cells were prepared from 2 healthy donors and co-cultured with primary hepatocytes from 2 HLA-A*02:01^{+} adults.

Figure S3

A

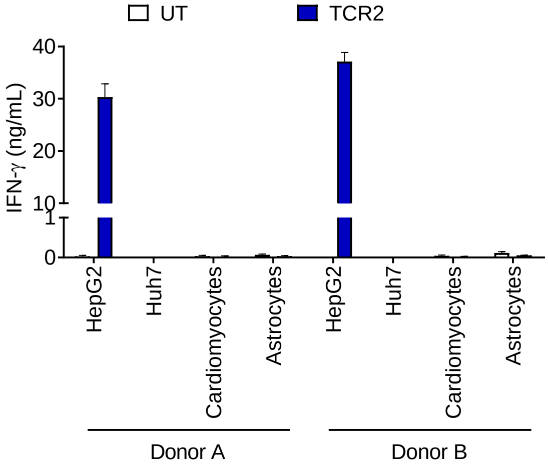

B

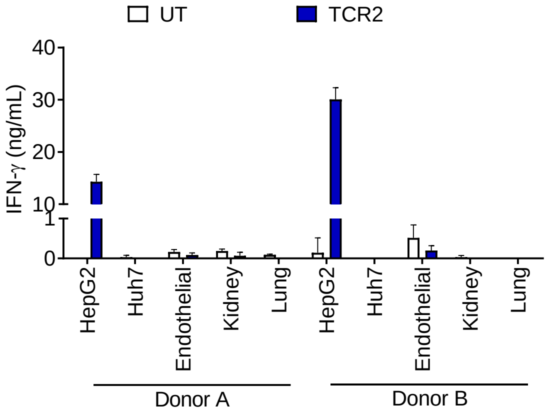

Figure S3. AFP TCR 2 displays no reactivity towards HLA-A*02:01^{+} primary or iCell-derived cells representing essential organs. (**A**, **B**) IFN-γ concentration in the co-cultured supernatant from T cells transduced with AFP TCR 2 or the UT with the indicated HLA-A*02:01^{+} primary cells. IFN-γ in the supernatant was measured by ELISA. T cells were prepared from 2 healthy donors (Donor A and Donor B) and co-culture with iCell derived cardiomyocytes and astrocytes (**A**) and endothelial cells, as well as primary lung and kidney epithelial cells (**B**). Data is presented as mean + s.d. of quadruplicate co-cultures. HepG2 and Huh7 cells were included as positive and negative controls, respectively.

Figure S4

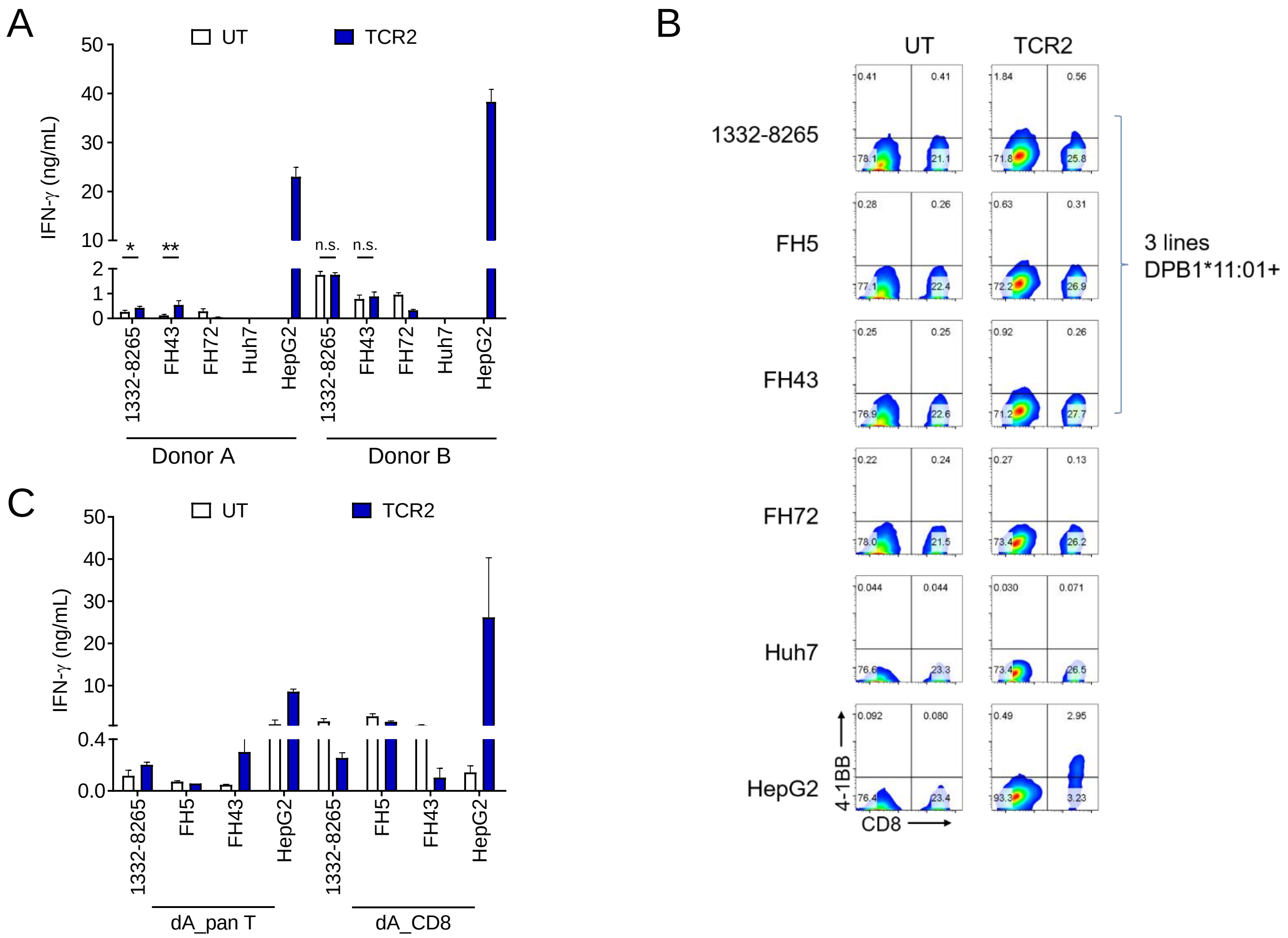

Figure S4. AFP TCR 2 displayed marginal alloreactivity towards HLA-DPB1*11:01. (**A**) IFN-γ concentration in the supernatant of T cells transduced with AFP TCR 2 or the UT co-cultured with the indicated Epstein-Barr virus transformed B cell lines. IFN-γ was measured by ELISA. T cells were prepared from 2 healthy donors (Donor A and Donor B). 1332-8265 and FH43 share a class II HLA DPB1*11:01 that is negative for FH72. Data are presented as mean + s.d. of quadruplicate co-cultures. HepG2 and Huh7 cells were included as positive and negative controls, respectively. (**B**) 4-1BB expression on T cells prepared as in **A**. T cells were then analyzed by FACS for surface expression of 4-1BB on total $CD3^+$ UT or AFP TCR 2 transduced T cells (gated on mouse $TCR\beta^+$). In addition to 1332-8265 and FH43, FH5 is another B cell line in the panel of 38 lines that carries class II HLA DPB1*11:01. (**C**) IFN-γ concentration in the co-cultured supernatant from total pan T cells or $CD8^+$ cells isolated from Donor A (dA), and transduced with AFP TCR 2 or left untransduced (UT), and then co-cultured overnight with the indicated B cell lines. IFN-γ was measured by a bead-based immunoassay run on a FACS instrument. Data are presented as mean + s.d. * = *P* < 0.05; ** = *P* < 0.01; n.s. = not significant (unpaired two-tailed Student's t-test).

Table S1: Allo-reactivity panel of 38 EBV transformed B cell lines

Cell Line Name	Gender	Ethnic origin	A	B	Cw
1332-4889	M	Caucasian	0301 0301	3501 3501	0401 0401
1332-8257	F	Caucasian	0301 3001	3501 0702	0401 0702
1332-8265	F	Caucasian	0301 0301	5601 3501	0102 0401
1333-8280	M	Caucasian	0301 0101	3501 5801	0401 0701
1344-8322	M	Caucasian	2402 0101	4001 1501	0304 0303
1413-1218	F	Caucasian	6801 1101	1302 0702	0303 0702
AMAI	M	Algerian	6802	5301	0401
DUG150		Unknown	02 6801	5802 4501	0602 1601
FH43	M	American Black	3001 3301	5301 8101	04 08
FH5	M	Caucasian	0201 2902	2709 4403	0102 1601
FH69			0203 11	52 5801	1203 03
FH72			01 6802	44 4405	0102 0202
FH9	M	Other	2402 3303	4801 4403	0801 0701
KAS011	F	Yugoslavian	0101	3701	0602
KT12	M	Japanese	2402 3101	3501 5201	0401 1202
KT14	M	Japanese	2402 2602	4006 5101	0801 1402
LATIF	M	Asian Indian	3101 1101	5101 1508	1602 0102
LKT3	M	Japanese	2402 2402	5401	0102
LUY	F	Dutch	0201	5101	1402
MANIKA	F	Tamil Asian Indi	0301	5001	0602
SCL-116A	F	Caucasian	0301 3002	1801 5601	0102 05
T7526	M	Chinese	0206 0207	4601	0102 0801
TAB	M	Japanese	0207 0201	4601	0102
TUBO	F	French	0301 0216	5101	1502 0704
LBF	M	English Caucas	3001 3001	1302 1302	0602
WUZH1		Chinese	0253N 1101	3701 5801	0302 0602
DEM		German	0201	5701	0602
KT17	F	Japanese	0206 1101	1501 3501	0303 0401
FH35	M	Filipino	3401 3401	15 1502	0303 04
FH28	F	Caucasian	1101 0101	2704 0801	0801 0701
280599		Asian	2402 2604	3802 3901	0702 0702
LUCE			1101 0201	3503 2702	1203 0202
1346-8358	M	Caucasian	0206 2601	3501 3801	0401 1203
FH10	F	Other	0206 2402	5502 4002	0102 1502
FH56	F	Other	0205 0301	49 4102	07 04
1416-1337	M	Caucasian	0205 2501	4901 4402	0701 0501
FH19		Chinese	0203 3401	3802 15	07 04
LCK		Asian	0203 1102	4601 3802	0702 1202
