# Supplementary material for: Selection of a Clinical Lead TCR Targeting Alpha-Fetoprotein-Positive Liver Cancer Based on a Balance of Risk and Benefit
Source: Front Immunol. 2020 Apr 27;11:623. doi: 10.3389/fimmu.2020.00623 (PMC7203609; doi:10.3389/fimmu.2020.00623)
Supplement: Supplementary file 2 [file Data_Sheet_2.PDF]

Table S2 Frequencies of HLA subtypes covered by the allo-reactivity panel among different ethnic groups

USA NMDP European Caucasian (n=1242890)					
**Allele**	**Freq.**	**Allele**	**Freq.**	**Allele**	**Freq.**
A*11:01	0.061	B*40:01	0.053	C*07:02	0.141
A*24:02	0.085	B*46:01	0.00008	C*01:02	0.034
A*02:01	0.276	B*13:02	0.024	C*06:02	0.093
A*33:03	0.00321	B*51:01	0.047	C*03:04	0.075
A*02:07	0.00004	B*15:01	0.061	C*08:01	0.0004
A*30:01	0.013	B*58:01	0.00726	C*03:03	0.053
A*02:06	0.00182	B*40:06	0.00032	C*04:01	0.106
A*31:01	0.027	B*54:01	0.00004	C*03:02	0.0022
A*01:01	0.165	B*48:01	0.0007	C*14:02	0.013
A*03:01	0.14	B*52:01	0.00886	C*15:02	0.022
A*26:01	0.031	B*44:03	0.047	C*12:02	0.0087
A*02:03	0.00002	B*35:01	0.056	C*12:03	0.049
A*11:02	0.00001	B*15:02	0	C*07:04	0.015
A*68:01	0.032	B*07:02	0.131	C*02:02	0.044
A*02:05	0.00966	B*40:02	0.013	C*05:01	0.094
A*33:01	0.0081	B*38:02	0.00004	C*07:01	0.16
A*34:01	0.00004	B*39:01	0.011	C*16:02	0
A*25:01	0.021	B*55:02	0.00003	C*16:01	0
A*29:02	0.035	B*37:01	0.014	**sum**	0.9103
A*02:53N	0	B*57:01	0.036		
A*30:02	0.009	B*27:04	0.00001		
A*26:02	0.00001	B*35:03	0.016		
A*68:02	0.00838	B*08:01	0.114		
A*02:16	0	B*44:02	0.095		
A*26:04	0	B*56:01	0.00643		
**sum**	0.92629	B*50:01	0.011		
		B*38:01	0.021		
		B*18:01	0.044		
		B*49:01	0.016		
		B*53:01	0.00339		
		B*45:01	0.00589		
		B*81:01	0.00008		
		B*27:02	0.00393		
		B*15:08	0.00011		
		B*41:02	0.00496		
		B*44:05	0.00286		
		B*27:09	0.00006		
		B*58:02	0.00015		
		**sum**	0.8552		

USA NMDP African American pop 2 (n=416581)					
**Allele**	**Freq.**	**Allele**	**Freq.**	**Allele**	**Freq.**
A*11:01	0.014	B*40:01	0.013	C*07:02	0.071
A*24:02	0.025	B*46:01	0.00007	C*01:02	0.0078
A*02:01	0.123	B*13:02	0.00782	C*06:02	0.087
A*33:03	0.052	B*51:01	0.022	C*03:04	0.057
A*02:07	0.00002	B*15:01	0.011	C*08:01	0.0011
A*30:01	0.068	B*58:01	0.038	C*03:03	0.013
A*02:06	0.00071	B*40:06	0.00041	C*04:01	0.204
A*31:01	0.01	B*54:01	0.00003	C*03:02	0.018
A*01:01	0.047	B*48:01	0.00045	C*14:02	0.017
A*03:01	0.084	B*52:01	0.015	C*15:02	0.0054
A*26:01	0.015	B*44:03	0.046	C*12:02	0.0012
A*02:03	0.00016	B*35:01	0.069	C*12:03	0.015
A*11:02	0.00001	B*15:02	0.00031	C*07:04	0.0072
A*68:01	0.04	B*07:02	0.073	C*02:02	0.089
A*02:05	0.015	B*40:02	0.00334	C*05:01	0.034
A*33:01	0.021	B*38:02	0.00007	C*07:01	0.117
A*34:01	0.00008	B*39:01	0.00316	C*16:02	0.0002
A*25:01	0.00344	B*55:02	0.00007	C*16:01	0.097
A*29:02	0.032	B*37:01	0.0055	**sum**	0.8417
A*02:53N	0	B*57:01	0.00711		
A*30:02	0.067	B*27:04	0.00002		
A*26:02	0.00001	B*35:03	0.00198		
A*68:02	0.06	B*08:01	0.038		
A*02:16	0.00003	B*44:02	0.021		
A*26:04	0	B*56:01	0.00234		
**sum**	0.67746	B*50:01	0.00891		
		B*38:01	0.00219		
		B*18:01	0.032		
		B*49:01	0.028		
		B*53:01	0.118		
		B*45:01	0.05		
		B*81:01	0.02		
		B*27:02	0.00036		
		B*15:08	0.00004		
		B*41:02	0.00562		
		B*44:05	0.00029		
		B*27:09	0.00001		
		B*58:02	0.042		
		**sum**	0.6861		

USA NMDP Hispanic South or Central American					
**Allele**	**Freq.**	**Allele**	**Freq.**	**Allele**	**Freq.**
A*11:01	0.046	B*40:01	0.014	C*07:02	0.121
A*24:02	0.132	B*46:01	0.00023	C*01:02	0.054
A*02:01	0.209	B*13:02	0.013	C*06:02	0.061
A*33:03	0.00818	B*51:01	0.061	C*03:04	0.06
A*02:07	0.00006	B*15:01	0.027	C*08:01	0.015
A*30:01	0.02	B*58:01	0.014	C*03:03	0.03
A*02:06	0.02	B*40:06	0.00134	C*04:01	0.176
A*31:01	0.044	B*54:01	0.00009	C*03:02	0.0049
A*01:01	0.073	B*48:01	0.014	C*14:02	0.015
A*03:01	0.074	B*52:01	0.021	C*15:02	0.039
A*26:01	0.03	B*44:03	0.055	C*12:02	0.012
A*02:03	0.00016	B*35:01	0.071	C*12:03	0.042
A*11:02	0.00001	B*15:02	0.00029	C*07:04	0.0055
A*68:01	0.048	B*07:02	0.058	C*02:02	0.037
A*02:05	0.015	B*40:02	0.048	C*05:01	0.058
A*33:01	0.022	B*38:02	0.00023	C*07:01	0.102
A*34:01	0.00019	B*39:01	0.0073	C*16:02	0.0039
A*25:01	0.01	B*55:02	0.00009	C*16:01	0.05
A*29:02	0.044	B*37:01	0.00749	**sum**	0.8864
A*02:53N	0	B*57:01	0.015		
A*30:02	0.027	B*27:04	0.00003		
A*26:02	0.00002	B*35:03	0.015		
A*68:02	0.025	B*08:01	0.039		
A*02:16	0	B*44:02	0.04		
A*26:04	0	B*56:01	0.00344		
**sum**	0.84762	B*50:01	0.015		
		B*38:01	0.02		
		B*18:01	0.041		
		B*49:01	0.026		
		B*53:01	0.02		
		B*45:01	0.017		
		B*81:01	0.00229		
		B*27:02	0.0019		
		B*15:08	0.00048		
		B*41:02	0.00516		
		B*44:05	0.0013		
		B*27:09	0.00002		
		B*58:02	0.0037		
		**sum**	0.67938		

USA NMDP Mexican or Chicano (n=261235)					
**Allele**	**Freq.**	**Allele**	**Freq.**	**Allele**	**Freq.**
A*11:01	0.048	B*40:01	0.014	C*07:02	0.137
A*24:02	0.13	B*46:01	0.0002	C*01:02	0.051
A*02:01	0.223	B*13:02	0.014	C*06:02	0.06
A*33:03	0.00493	B*51:01	0.058	C*03:04	0.073
A*02:07	0.00002	B*15:01	0.031	C*08:01	0.036
A*30:01	0.016	B*58:01	0.00901	C*03:03	0.037
A*02:06	0.05	B*40:06	0.0013	C*04:01	0.171
A*31:01	0.053	B*54:01	0.00006	C*03:02	0.0037
A*01:01	0.074	B*48:01	0.025	C*14:02	0.0096
A*03:01	0.081	B*52:01	0.026	C*15:02	0.036
A*26:01	0.026	B*44:03	0.047	C*12:02	0.011
A*02:03	0.00012	B*35:01	0.08	C*12:03	0.039
A*11:02	0.00001	B*15:02	0.0003	C*07:04	0.0048
A*68:01	0.052	B*07:02	0.057	C*02:02	0.034
A*02:05	0.014	B*40:02	0.057	C*05:01	0.057
A*33:01	0.02	B*38:02	0.00034	C*07:01	0.092
A*34:01	0.00037	B*39:01	0.00815	C*16:02	0.0038
A*25:01	0.011	B*55:02	0.00009	C*16:01	0.047
A*29:02	0.039	B*37:01	0.0073	**sum**	0.9028
A*02:53N	0	B*57:01	0.013		
A*30:02	0.023	B*27:04	0.00001		
A*26:02	0.00002	B*35:03	0.013		
A*68:02	0.018	B*08:01	0.042		
A*02:16	0	B*44:02	0.041		
A*26:04	0	B*56:01	0.0041		
**sum**	0.88347	B*50:01	0.013		
		B*38:01	0.018		
		B*18:01	0.041		
		B*49:01	0.023		
		B*53:01	0.012		
		B*45:01	0.014		
		B*81:01	0.00126		
		B*27:02	0.00195		
		B*15:08	0.00007		
		B*41:02	0.00416		
		B*44:05	0.00109		
		B*27:09	0.00002		
		B*58:02	0.00244		
		**sum**	0.68085		

USA NMDP South Asian Indian (n=185391)

Allele	Freq.	Allele	Freq.	Allele	Freq.
A*11:01	0.14	B*40:01	0.022	C*07:02	0.108
A*24:02	0.136	B*46:01	0.00102	C*01:02	0.035
A*02:01	0.049	B*13:02	0.018	C*06:02	0.139
A*33:03	0.099	B*51:01	0.075	C*03:04	0.016
A*02:07	0.0006	B*15:01	0.016	C*08:01	0.028
A*30:01	0.017	B*58:01	0.042	C*03:03	0.015
A*02:06	0.018	B*40:06	0.091	C*04:01	0.136
A*31:01	0.033	B*54:01	0.00027	C*03:02	0.042
A*01:01	0.155	B*48:01	0.00368	C*14:02	0.034
A*03:01	0.064	B*52:01	0.075	C*15:02	0.108
A*26:01	0.042	B*44:03	0.074	C*12:02	0.081
A*02:03	0.011	B*35:01	0.062	C*12:03	0.049
A*11:02	0.00012	B*15:02	0.026	C*07:04	0.015
A*68:01	0.068	B*07:02	0.044	C*02:02	0.0093
A*02:05	0.00998	B*40:02	0.00296	C*05:01	0.0084
A*33:01	0.00124	B*38:02	0.00693	C*07:01	0.104
A*34:01	0.00014	B*39:01	0.00548	C*16:02	0.028
A*25:01	0.00013	B*55:02	0.00049	C*16:01	0.0007
A*29:02	0.00129	B*37:01	0.034	**sum**	0.9564
A*02:53N	0	B*57:01	0.068		
A*30:02	0.00243	B*27:04	0.00198		
A*26:02	0.00003	B*35:03	0.072		
A*68:02	0.00028	B*08:01	0.037		
A*02:16	0.00301	B*44:02	0.00816		
A*26:04	0	B*56:01	0.00608		
**sum**	0.85125	B*50:01	0.017		
		B*38:01	0.0028		
		B*18:01	0.025		
		B*49:01	0.00614		
		B*53:01	0.00126		
		B*45:01	0.00088		
		B*81:01	0.00005		
		B*27:02	0.00077		
		B*15:08	0.00391		
		B*41:02	0.00058		
		B*44:05	0.00004		
		B*27:09	0.00001		
		B*58:02	0.00006		
		**sum**	0.85155		
